# Analytical reactivity of 13 commercially available rapid influenza diagnostic tests with H3N2v and recently circulating influenza viruses

**DOI:** 10.1111/irv.12246

**Published:** 2014-04-03

**Authors:** Michael E Bose, Amy Sasman, Hong Mei, Kate C McCaul, William J Kramp, Li-Mei Chen, Roxanne Shively, Tracie L Williams, Eric T Beck, Kelly J Henrickson

**Affiliations:** a Medical College of WisconsinMilwaukee, WI, USA; b Biomedical Advanced Research and Development Authority, Office of the Assistant Secretary for Preparedness and Response, US Department of Health and Human ServicesWashington, DC, USA; c Influenza Division, Centers for Disease Control and PreventionAtlanta, GA, USA; d Division of Laboratory Science, National Center for Environmental Health, Centers for Disease Control and PreventionAtlanta, GA, USA; e Dynacare LaboratoriesMilwaukee, WI, USA; f Children's Research InstituteWauwatosa, WI, USA

**Keywords:** Diagnostic, FDA, H3N2v, influenza, rapid

## Abstract

**Objectives:**

Rapid influenza diagnostic tests (RIDTs) used widely in clinical practice are simple to use and provide results within 15 minutes; however, reported performance is variable, which causes concern when novel or variant viruses emerge. This study's goal was to assess the analytical reactivity of 13 RIDTs with recently circulating seasonal and H3N2v influenza viruses, using three different viral measures.

**Design:**

Virus stocks were characterized by infectious dose (ID_50_) and nucleoprotein (NP) concentration, diluted at half-log dilutions, and tested with each RIDT and real-time RT-PCR.

**Results:**

Strong correlation was observed between NP concentration and RIDT reactivity; however, only weak correlation was seen with ID_50_ or *C*_t_ values. Only four RIDTs detected viral NP at the lowest dilution for all influenza A viruses (IAV). Influenza A viruses not detected by more than one RIDT had lower NP levels. Of the 13 RIDTs, 9 had no significant differences in reactivity across IAV when compared to NP levels.

**Conclusions:**

Previous reports of RIDT performance typically compare reactivity based on ID_50_ titers, which in this study correlated only weakly with proportional amounts of viral NP in prepared virus samples. In the context of the strong correlation of RIDT reactivity with NP concentration, H3N2v was found to be as reactive as seasonal circulating IAV. While these findings may not reflect clinical performance of these RIDTs, measuring NP concentration can be useful in the future to assess comparable reactivity of available RIDTs, or to assess reactivity with newly evolving or emerging viruses.

## Introduction

Rapid influenza diagnostic tests (RIDTs) are commonly used in clinical practice because they are simple to use and can provide results within 15 minutes. All RIDTs available in the USA during the 2012–13 season utilize lateral flow immunoassays with antibodies specific to the nucleoprotein of influenza A viruses (IAV) and influenza B viruses (IBV) for the rapid qualitative detection of each virus type. Currently available RIDTs rely on a visual colorimetric signal or require a reader to interpret reflectance or fluorescence. Previous reports note disparities between ID_50_ titers and RIDT reactivity with viral nucleoproteins from seasonal, swine, and avian IAV,^1^,^2^ while another report observed that low NP levels as measured by mass spectrometry were associated with reduced ranges of analytical reactivity for pandemic H1N1 (pH1N1) and human seasonal H3N2 viruses.^3^ With the emergence of the pH1N1 virus in humans, there was concern with the ability of available RIDTs to reliably detect this virus. During the early pandemic, RIDTs were reported to have reduced sensitivity, while later studies suggested otherwise.^4^–^7^

In 2011, an influenza A variant virus was sporadically detected in human respiratory specimens. This variant carries the matrix gene from pH1N1 and the remaining genes from a triple reassortant North American swine H3N2 virus.^8^,^9^ While the total number of reported cases of H3N2 variant (H3N2v) in 2011 was low with only 12 cases, the 309 cases reported in 2012 and continued cases in 2013 (http://www.cdc.gov/flu/swineflu/h3n2v-case-count.htm) raise concerns that this virus could spread more broadly in communities.^8^,^10^,^11^ As with the emergence of the pH1N1 virus, there are reports that some RIDTs may have reduced sensitivity for H3N2v,^1^,^12^ when measured against ID_50_ titer.

This study applied ID_50_, cycle threshold (*C*_t_) values, and nucleoprotein (NP) measures of virus stock dilutions to evaluate the reactivity ranges of 13 FDA-cleared RIDTs with a selection of seasonal and H3N2v viruses.

## Materials and methods

### Viruses

**Figure 1:**
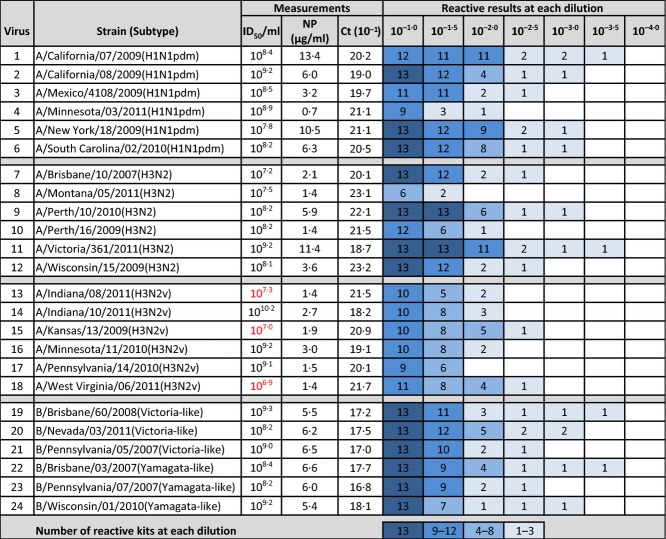
Viruses used in this study, with measurements by TCID_50_/ml or EID_50_/ml, NP in μg/ml as determined by mass spectrometry, and the *C*_t_ value of the 10^−1^ dilution. Viruses 13, 15, and 18 were quantified by TCID_50_/ml (red numbering). All others were quantified by EID_50_/ml. Also shown is the number of reactive rapid influenza diagnostic tests results (at least 2/3 positive) for each influenza virus dilution. A maximum of 13 test kits could be positive for each dilution.

Virus designations with stock concentrations are listed in Figure1. Frozen aliquots of stocks quantified by chicken embryo infectious virus titer (EID_50_/ml) or MDCK tissue culture (TCID_50_/ml) received from the Influenza Division, WHO Collaborating Center for Surveillance, Epidemiology and Control of Influenza, Centers for Disease Control and Prevention, Atlanta, GA, USA (CDC), were used for all determinations.

### Mass spectrometry

NP concentration (μg/ml) was measured by isotope dilution mass spectrometry as described for hemagglutinin and neuraminidase proteins.^13^,^14^ Virus stocks were enzymatically digested with trypsin and spiked with ^13^C- and ^15^N-labeled analogs of the NP target peptides (LIQNSITIER, LIQNSITIEK, and LIQNSLTIER for IAV and ALVDQVIGSR, VVLPISIYAK, and SGATGVAIK for IBV). Reverse-phase separation of peptides and analysis by mass spectrometry were as described.^13^,^14^ Publication with complete details of this method and applicability to a broader range of viruses is in process. Mass spectroscopy analysis was performed at the Division of Laboratory Science, National Center for Environmental Health, CDC.

### Virus dilution

One virus stock was used each day with the real-time RT-PCR and all RIDTs described in Table1. Each morning, a single virus stock was thawed and diluted in 0·9% saline (Sigma-Aldrich Company, St. Louis, MO), the only liquid medium compatible with all RIDTs used in this study. Virus stocks were thawed on ice and diluted in serial half-log-dilutions from 10^−1^ to 10^−4^. Each virus dilution was transferred into 200 μl aliquots and stored on ice or in the 4°C refrigerator until used that day.

**Table 1:** Reactivity of 13 FDA-approved rapid influenza diagnostic tests (RIDTs) with 24 recently isolated influenza viruses at any concentration

RIDT	No. of viruses reactive at any concentration				Viruses not reactive	Total no. of tests	No. of invalid tests	No. of false positives

	Flu A – H1N1pdm	Flu A – H3N2	Flu A – H3N2v	Flu B				
Sofia Influenza A+B FIA	6/6	6/6	6/6	6/6	–	513	4	1
BD Veritor Flu A+B – for swab specimens	6/6	6/6	6/6	6/6	–	444	0	0
X/pect® Flu A&B	6/6	5/6	6/6	6/6	8	420	0	0
OSOM Influenza A&B	6/6	5/6	6/6	6/6	8	417	0	0
Alere Influenza A&B	6/6	5/6	6/6	6/6	8	357	0	0
Directigen EZ Flu A+B	6/6	6/6	6/6	6/6	–	378	0	0
BD Veritor Flu A+B – for liquid specimens	6/6	6/6	6/6	6/6	–	381	6	0
TRUFLU	5/6	5/6	6/6	6/6	4, 8	411	0	0
3M™ Rapid Detection Flu A+B	5/6	5/6	5/6	6/6	4, 8, 13	366	0	0
QuickVue Influenza A+B	6/6	6/6	2/6	6/6	14, 15, 16, 17	357	0	0
BinaxNOW Influenza A & B	6/6	5/6	4/6	6/6	8, 13, 17	357	0	0
Status Flu A + B	4/6	6/6	1/6	6/6	3, 4, 14, 15, 16, 17, 18	405	0	4
SAS FluAlert Influenza A; SAS FluAlert Influenza B	3/6	4/6	0/6	6/6	1, 3, 4, 8, 10, 13, 14, 15, 16, 17, 18	264	0	0

### Real-time RT-PCR

The CDC Influenza Virus rRT-PCR Diagnostic (Flu A&B) Panel (Influenza Reagent Resource, Manassas, VA, USA) was performed on each dilution as previously described.^15^ XY scatter plots of log_10_ dilution versus the corresponding *C*_t_ value were generated (Microsoft Excel 2010, Microsoft Corp., Redmond, WA, USA). All 24 viral dilution series had a linear regression *r*^2^ value above 0·95 (generally >0·99), assuring consistent dilution series for each virus. The *C*_t_ values for the 10^−1^ dilution are used in analyses (see Figure1), as dilution curves tended to deviate from linearity when *C*_t_ values from the undiluted virus stock were included in the regression (data not shown).

### Rapid influenza diagnostic tests

**Figure 2:**
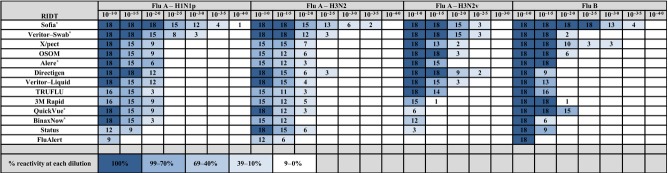
Reactivity of each rapid influenza diagnostic tests across influenza virus groups. Six viruses in each group were tested at three replicates per dilution for a maximum of 18 positive results per dilution. CLIA-waived tests are marked with an *.

Testing with RIDTs and RT-PCR was performed between October and December 2012 at the Medical College of Wisconsin. Rapid influenza diagnostic tests are listed in Figure2. Complete detailing of these RIDTs is available at http://www.cdc.gov/flu/professionals/diagnosis/clinician_guidance_ridt.htm#Table 2. Five of the RIDTs are CLIA-waived, categorized as simple laboratory examinations that have an insignificant risk of an erroneous result.

Positive and negative controls provided with each RIDT kit were tested upon receipt for each lot in every shipment. All aspects of the evaluation including diluent, swabs, and virus input were standardized. The manufacturer's instructions for testing a swab specimen directly (without placing the swab in transport medium) were always followed (nasopharyngeal swab instructions were used for most RIDTs; throat swab instructions for the BD Directigen EZ Influenza A+B).

Following virus stock dilution, 50 μl of each dilution was placed into three 1·5-ml microcentrifuge tubes and held on ice. A sterile foam swab (Catalog # 25-1506-1PF, Puritan Medical Products Co. LLC, Guilford, ME, USA) was used to absorb each of the 50 μl samples in the microcentrifuge tubes and used as the input. Adjustments to this procedure were used when RIDT instructions required input with liquid suspensions of swab samples. For the 3M™ Rapid Detection Flu A+B test and BD Veritor for Liquid Samples, after absorbing the sample the swab was placed into a tube containing 1 ml of UTM (Quidel Corporation, San Diego, CA, USA) and mixed prior to using the manufacturers' specified volume input for these two RIDTs. BinaxNOW requires placing the swab in an elution solution (either purchased or substituted with 500 μl saline, used in this testing). Both FluAlert (SA Scientific, San Antonio, TX, USA) RIDTs are only indicated for nasal wash and aspirate samples. As the required sample input for this RIDT is 250 μl, we combined the 50 μl dilution sample with 200 μl of 0·9% saline. Even though this is a CLIA-waived test, the moderate complexity protocol was used due to multiple invalid results during quality control testing with the waived protocol. Kit-provided flocked swabs were used with the Status® Flu A+B test (Princeton Biomedical, Monmouth Junction, NJ, USA), as instructions do not allow for foam swabs used with other RIDTs.

After the study started, Quidel issued a recall of previously used Sofia FIA lots. At that point, 1 false-positive influenza B result was recorded with a negative control during the use of over 450 Quidel Sofia tests (Table1). The manufacturer replaced remaining kits and no further influenza B false positives occurred with replacement kits. With the Status RIDT, four false positives for influenza B were observed: two in negative control replicates, one in a 10^−1·5^ dilution of an A/Minnesota/03/2011 replicate, and one in a 10^−1^ dilution of an A/Victoria/361/2011 replicate.

### Statistical analyses

For analyses, the highest dilution reactive (HDR) was determined as the one in which two of the three replicates were positive for any one virus. Spearman's rank correlation analyses between stock ID_50_ titers, NP concentration, and 10^−1^ dilution *C*_t_ for each virus were used to assess the associations between these measures. A Spearman's rank correlation was also performed comparing the mean HDR for all RIDTs to the stock ID_50_ titers, NP concentration, and 10^−1^ dilution *C*_t_ for each virus. TCID_50_ quantitations (three viruses) were omitted only from correlation analyses with ID_50_ due to unverified comparability of TCID_50_ and EID_50_ methods. Any virus and RIDT combination with no reactivity in the 10^−1^ dilution was not included in the correlation calculations; however, for subsequent analyses, the nominal value of the 10^−0·5^ dilution was used. To compare virus groups for each RIDT, a log transformation was applied to normalize the variances of the reactivity measures before performing one-way anovas. *P*-values between significantly different IAV groups for individual RIDTs were determined by Tukey's honest significant difference test. All analyses were performed in Microsoft Excel 2010.

## Results

### Comparison of ID_50_, Nucleoprotein, and RT-PCR *C*_t_ measures

Figure1 lists the ID_50_, *C*_t_ values, and NP concentration for the virus stocks. Correlation coefficients for the association between ID_50_, NP concentration, and *C*_t_ values across viruses were weak for ID_50_ and *C*_t_ versus NP (0·094 and −0·35) and moderate for ID_50_ versus *C*_t_ values (−0·75). When the viruses with TCID_50_ quantitation were included, the correlation was stronger for ID_50_ versus NP and was weaker for ID_50_ versus *C*_t_ values, but had no impact on interpretation of the results.

### RIDT results

Figure1 shows the number of tests that were positive in at least two of three replicates at each dilution for each of the 24 viruses. All 13 RIDTs were reactive with seven of the 18 IAVs and all six of the IBVs. Only four RIDTs (Sofia, both Veritors, Directigen) were reactive with all IAVs in the initial dilution (10^−1^) tested (Figure2 and Table1). The remaining RIDTs were not reactive with at least one IAV at any dilution tested. Notably, nine RIDTs detected all pH1N1 viruses, six RIDTs detected all H3N2 viruses, and eight RIDTs detected all H3N2v viruses in the 10^−1^ dilution. One RIDT (SAS FluAlert Influenza A test and Influenza B test, which are separate test units, but boxed together in one kit) had reactivity in only seven IAVs (none in the H3N2v group, three in the pH1N1, and four in the H3N2 seasonal group). The reactivity of other RIDTs ranged from detection of 11 (Status) to 17 (X/Pect, OSOM, Alere) IAVs in the 10^−1^ dilution (Table1).

For IAVs, patterns of reactivity are variable across all virus groups for both individual viruses (Figure1) and for individual RIDTs (Figure2). While we chose to score a dilution as reactive if 2 of 3 replicates at that dilution were positive, the majority of RIDTs yielded 3 of 3 positives at the highest dilution scored as reactive, and 0 of 3 positive results at all higher dilutions. There were seven occurrences for which an RIDT was scored reactive with 2 of 3 replicates positive. A total of 20 occurrences had only 1 of 3 positive replicates for any one RIDT in the next dilution beyond the HDR. The majority of these (15 of 20) were with RIDTs interpreted by automated readers from both fluorescent (Sofia, 3M) and reflectance (Veritor) signals. These readers may discriminate subtle differences in reaction intensity that are not apparent in visual reads.

Differences in mean stock NP concentrations between IAV that were reactive in all RIDTs and those that were not reactive in more than one RIDT suggest a link between NP concentration and test reactivity (Table S1). Those IAVs not reactive in more than one RIDT had a mean stock NP concentration of 1·9 μg/ml (range: 0·7–3·2), whereas the mean for all IAVs was 4·3 NP μg/ml (range: 0·7–13·4 μg/ml). ID_50_ titer ranges were similar and overlapped considerably across each of the virus groups as did the *C*_t_ value ranges at the 10^−1^ dilution for the IAVs. The range of stock NP concentrations was narrower for IBVs, yet HDRs varied widely across RIDTs.

**Figure 3:**
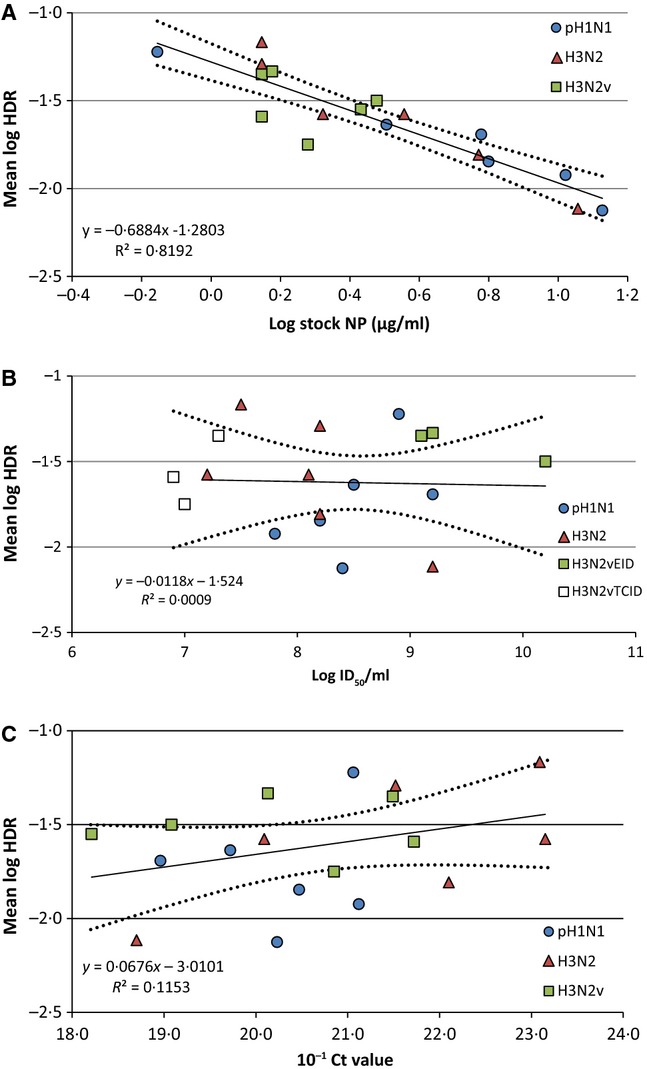
Scatter plots showing the mean log highest dilution reactive (HDR) across all rapid influenza diagnostic tests for each virus tested against (A) the log stock NP concentration, (B) the log stock ID_50_, and (C) the 10^−1^ dilution *C*_t_ value. The black line shows the linear regression trend line and the black dotted lines show the 95% confidence interval, along with equations and *R*^2^ values for each trend line. Only viruses quantified by EID_50_/ml were used for the trendline in B.

Figure3 shows the mean log HDR for each virus plotted versus stock log NP, stock log ID_50_, and 10^−1^ dilution *C*_t_ values. For IAV, the correlation is strong (−0·86) between stock NP concentration and the mean HDR for all test kits. On the other hand, correlation between stock ID_50_ and HDR is practically zero (−0·015) and weak between *C*_t_ values and HDR (0·24).

**Figure 4:**
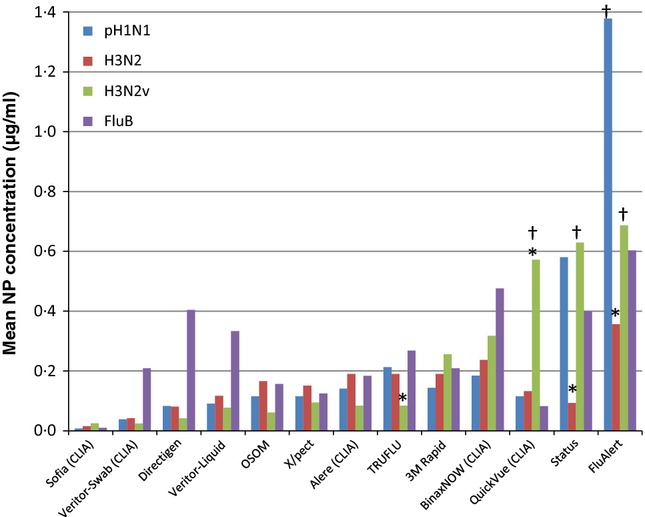
Graph showing the mean NP concentration at the highest dilution reactive for each of the virus groups (pH1N1, H3N2, H3N2v, and influenza B) for each of the rapid influenza diagnostic tests (RIDTs). * indicates that an influenza A viruses (IAV) group is significantly different from other IAV groups for that RIDT based on Tukey's HSD test. The QuickVue test was significantly less reactive with H3N2v than with pH1N1 and H3N2. The Status test was significantly more reactive with H3N2 than with pH1N1 or H3N2v. The Flu Alert test was significantly more reactive with H3N2 than with pH1N1 (H3N2v could not be statistically evaluated). The TRU FLU test was significantly more reactive with H3N2v than with pH1N1. A † represents situations in which an RIDT was reactive with 3 or less viruses in a group. CLIA indicates that an RIDT is CLIA-waived.

As NP concentration versus mean HDR had a strong association across all RIDTs for IAV, the mean NP (for all viruses in a virus group) was plotted against each RIDT (Figure4). anovas showed no significant difference (*P*-value >0·05) between any of the IAV groups for nine individual RIDTs. These nine RIDTs include those reactive with all IAVs (*n* = 4). While individual RIDTs showed some variation between IAVs, no individual IAV subtype was significantly less reactive across all RIDTs, when compared with NP concentrations by anova as shown in Figure4 with four exceptions. The FluAlert RIDT was apparently less reactive with pH1N1 and H3N2v (only 3 and 0, respectively) than with H3N2 viruses (four reactive). This particular RIDT, however, was less reactive for H3N2 than other RIDTs when compared to NP levels detected as shown in Figure4. Additionally, this RIDT failed to react with the pH1N1 virus with the highest stock NP measure (13·4 μg/ml for A/California/07/2009). Status was also less reactive with pH1N1 and H3N2v than with H3N2 viruses (4 and 1 reactive versus all 6, respectively) yet was similar to other RIDTs in the calculated NP reactivity for H3N2 viruses. QuickVue was less reactive with H3N2v than with either the pH1N1 or H3N2 group (only 2 of 6 H3N2v viruses were reactive, while all other IAVs were reactive). Note: Reduced reactivity with H3N2v viruses for QuickVue and FluAlert was also observed in a previous study.^1^ TRUFLU, on the other hand, was more reactive with the H3N2v group than with the pH1N1 group (6 reactive with H3N2v versus 5 with pH1N1) yet no statistical difference was found between pH1N1 and H3N2 virus groups for mean reactive NP levels. Figure4 notes IAV groups that were significantly more or less reactive (*P*-value <0·05) for any one RIDT.

Figure4 also shows the mean NP levels at HDR for each of the RIDTs with IBV. Trends or correlation for IBV were not evident as the virus NP stock concentrations (and also ID_50_ titers and *C*_t_ values) for this small group of viruses were notably uniform, yet HDRs varied widely across individual RIDTs.

## Conclusions

The primary goal of this study was to determine whether RIDTs are as reactive with H3N2v IAVs as with other influenza viruses. Aggregate reactivity results for all RIDTs with each virus in Figure1 suggest reduced reactivity with H3N2v when shown by dilution. However, further analysis referencing stock NP values supports that the majority of RIDTs were not less reactive with H3N2v virus NP than with other IAV-NP. In this evaluation, H3N2 and pH1N1 as well as H3N2v viruses with low stock NP concentrations were more likely to be non-reactive or to have reduced ranges of reactivity (and lower HDRs) in RIDTs. Notably, the H3N2v virus stocks had the lowest NP concentrations as a group, which could be a factor with reduced sensitivity in clinical practice if these viruses also produce less NP during human infection.

The strong correlation of the virus stock NP concentrations with mean HDRs in this evaluation is not unexpected given that all of the RIDTs are designed to detect viral nucleoprotein using different antibodies to capture and detect influenza A and B viruses, and this association was also observed in a previous study.^3^ The poor correlation of these IAV stock NP concentrations with ID_50_ titers, a virus measure known for variability between laboratories and methods, warrants caution with assessing RIDT analytical reactivity with propagated influenza viruses characterized solely by ID_50_ titers.

In this study, the BD Veritor RIDT for liquid specimens (e.g., swab in transport media) showed a decrease in reactivity when compared to the Veritor RIDT for swab specimens (tested directly). Previous testing with an RIDT using two sets of virus samples (50 μl adsorbed onto a swab and 50 μl added to 1 ml diluent prior to testing) showed a consistent decrease in reactivity for the set added to diluent (data not shown). A major advantage with RIDTs is the rapid time to results (if specimens are tested at the time of collection); placing swabs into transport media for RIDT may offset the benefit with rapid results if NP levels are lowered by dilution.

Although the correlation between NP concentrations and IAV reactivity is strong (*r*^2^ = −0·86) in this study, it is unable to explain all of the variability in reactivity due to other potentially contributing factors. Such factors can include proprietary differences between individual RIDTs, sequence variations affecting epitope-binding sites, or differences in virus replication properties within infected host or culture cells and the potential for multiple virus quasispecies. Observations from this study, specifically that the (A/Montana/05/2011) virus with the lowest NP concentration was not reactive in the least number of RIDTs and (A/California/07/2009) virus with the highest NP concentration was not reactive in all of the RIDTs, suggest that there must be factors other than NP concentration contributing to reactivity. Furthermore, several RIDTs showed significant differences in reactivity between IAV groups, suggesting that antibody design may not be optimal for all IAV and amino acid variations between IAV groups could be a factor. A previous report^1^ explored the phylogenetic relationship between variant IAVs and seasonal IAVs and suggested that amino acid changes in a target epitope region could hypothetically reduce reactivity.

These findings are limited by the use of specific viruses, propagated under conditions that can influence ID_50_ titers and relative *C*_t_ values of harvested influenza viruses, as well as NP levels. Only six individual viruses comprised each influenza virus group as representative of each type or subtype. The dependency of the strong correlation with NP levels on virus sourcing, growth conditions, and other stock propagation variables requires further research. In addition, the assumption that reactivity of a single RIDT should be consistent across IAV groups as long as antibody recognition and amounts of influenza NP are similar needs to be verified.

In conclusion, RIDTs are generally as reactive with H3N2v as with other IAVs even though differences in reactivity were observed between IAVs (seasonal or variant) for individual RIDTs. Our observation that H3N2v viruses as a group produced less NP in virus culture may be indicative of their growth in mammalian cells. Further characterization of these viruses in both virus culture and respiratory samples would be important for better understanding of these observations, for improving RIDT performance, and for use of RIDTs in clinical practice. This evaluation reinforces that negative RIDT results are more likely when any virus samples have low NP concentrations, regardless of ID_50_ titers or *C*_t_ values. Furthermore, performance estimates from either analytical or clinical studies may vary by the nature of the virus, as well as by specimen collection factors that optimize the amounts of viral NP sampled. Additional research is needed to determine ranges of NP concentrations in clinical specimens with different influenza A and B viruses or to verify that NP concentrations from an *in vitro* propagated virus reflect replication properties of the virus in host cells. Regardless, a standardized mass spectrometric method for directly measuring nucleoprotein levels in analytical virus preparations could offer an appropriate benchmark for comparing the reactivity of RIDTs, or assessing reactivity with newly evolving or emerging influenza viruses.

## Addendum

M. E. Bose contributed to data analysis and interpretation and manuscript preparation. A. Sasman contributed to the RIDT, data compilation, and manuscript preparation. H. Mei and K. McCaul performed the RIDT and data compilation. W. Kramp and R. Shively contributed to the concept and design of the study, data analysis and interpretation, and manuscript preparation. E. T. Beck and K. J. Henrickson contributed to the concept and design of the study and manuscript preparation. L. Chen prepared the viruses used in this study. T. L. Williams managed the isotope dilution mass spectrometry quantitation of NP for these viruses. All authors have approved the final version of the manuscript.

## Supporting Information

**Table S1.** Average measurement by virus group.
