# Supplementary material for: Analytical reactivity of 13 commercially available rapid influenza diagnostic tests with H3N2v and recently circulating influenza viruses
Source: Influenza Other Respir Viruses. 2014 Apr 3;8(4):474–81. doi: 10.1111/irv.12246 (PMC4181808; doi:10.1111/irv.12246)
Supplement: Supplementary file 1 — Table S1. Average measurement by virus group. [file irv0008-0474-SD1.docx]

| **Supplemental Table 1. Average Measurement by Virus Group.** | | | |
| --- | --- | --- | --- |
| **Virus Group** | **ID_50_/ml** | **NP (µg/ml)** | **Ct Value** |
| pH1N1 | 10^8.7^ (10^7.8^ – 10^9.2^) | 6.7 (0.7 - 13.4) | 20.3 (19 – 21.1) |
| H3N2 | 10^8.5^ (10^7.2^ – 10^9.2^) | 4.3 (1.4 - 11.4) | 21.5 (18.7 – 23.2) |
| H3N2v | 10^9.5^ (10^6.9^ – 10^10.2^) | 2.0 (1.4 - 3) | 20.3 (18.2 – 21.7) |
| All Flu A | 10^9.1^ (10^6.9^ – 10^10.2^) | 4.3 (0.7 - 13.4) | 20.7 (18.2 – 23.2) |
| All Flu B | 10^8.9^ (10^8.2^ – 10^9.3^) | 6.0 (5.4 - 6.6) | 17.7 (16.8 – 18.1) |
| Reactive in All RIDTs | 10^8.8^ (10^6.9^ – 10^9.3^) | 6.3 (2.1 - 11.4) | 19.2 (16.8 – 23.2) |
| Not Reactive in More than One RIDT | 10^9.3^ (10^6.9^ – 10^10.2^) | 1.9 (0.7 - 3.2) | 20.6 (18.2 – 23.1) |
